# Editorial: Decoding muscle asymmetry: insights into performance and injury prevention in sports

**DOI:** 10.3389/fspor.2026.1860680

**Published:** 2026-05-07

**Authors:** Lazar Toskić, Saša Đurić, Francisco Pradas de la Fuente

**Affiliations:** 1 Faculty of Sport and Physical Education, University of Priština-Kosovska Mitrovica, Leposavić, Serbia; 2 Faculty of Sport, University ‘”Union-Nikola Tesla’’, Belgrade, Serbia; 3 Liberal Arts Department, American University of the Middle East, Egaila, Kuwait; 4 Faculty of Health and Sports Sciences, University of Zaragoza, Huesca, Spain

**Keywords:** injury risk, muscle asymmetry, performance, rehabilitation, sports science

Muscle asymmetry, the imbalance between muscle groups or sides of the body in various properties such as strength, power, muscle mass, and electrical activity, has become an important and frequently investigated topic. This interest stems from the fact that muscle asymmetry could potentially decrease sports performance and increase the risk of injuries. The special issue “*Decoding Muscle Asymmetry: Insights into Performance and Injury Prevention in Sports”* aimed to advance understanding of muscle asymmetry by integrating insights from sports science and medicine, with a primary focus on exploring its multifaceted aspects, including strength, power, balance, flexibility, stiffness, electrical activity, range of motion, muscle mass, and kinematics. Additionally, the issue sought to elucidate the relationship between muscle asymmetry and various factors such as sports performance, success, injury risk, rehabilitation, health status, and overall quality of life, with the overarching goal of fostering a more nuanced appreciation of muscle asymmetry's role in optimizing sports performance and mitigating injuries.

To accomplish these aims, the special issue included original articles, case reports, and perspective articles mainly from the fields of sports training and technique, as well as musculoskeletal disorders and sports diagnostics. In the area of sports training and technique, Muehlbauer et al. examined the impact of unilateral single-mode balance training vs. combined balance and plyometric training on soccer players' interlimb asymmetry in balance and neuromuscular performance. The interventions consisted of balance exercises for the single-mode balance training group, balance and plyometric exercises for the combined training group, and stretching exercises for the active control group. The results showed that nine weeks of unilateral single-mode balance training and combined training were effective and feasible (attendance rates of ≈ 99%–100%). In particular, unilateral single-mode balance training was suitable for reducing the limb symmetry index value of the Y Balance Test – Lower Quarter anterior reach, a predictor for time-loss non-contact lower limb injuries. Ye et al. investigated the influence of arch height on isokinetic knee muscle strength at varying angular velocities, providing a potential scientific basis for the prevention and rehabilitation of associated conditions. Their results showed that foot arch height is associated with knee muscle strength at varying angular velocities. Individuals with low arches exhibited greater muscle strength in the posterior segments of the knee range at lower velocities, while those with abnormal arch heights demonstrated weaker knee muscle strength at high velocities. These findings suggest that individuals with abnormal arch heights may benefit from targeted high-speed knee exercise training to enhance muscle performance and mitigate injury risk in competitive sports. Stefansdottir et al. evaluated asymmetries in leg lean mass, dynamic balance, landing kinetics, and postural stability among 12-year-old female soccer players. Leg lean mass was assessed via dual-energy x-ray absorptiometry (DXA), dynamic balance using the Y Balance Test (YBT), and single-leg landing test with VALD force plates. No significant asymmetries were found in leg lean mass, landing force, or time to stabilization. However, significant asymmetry was observed in the anterior reach on the YBT, favouring the non-preferred kicking leg. No differences were seen in other YBT directions. These results suggest that asymmetries in young female players may begin to emerge in specific functional domains, even in the absence of structural or kinetic differences. Another study examined the influence of asymmetry on movement performance. Namely, Horiguchi et al. investigated the effects of foot strike patterns on plantar pressure distribution during uphill, level, and downhill running. Eleven participants performed six randomized treadmill trials running at 3.33 m/s, combining two foot strike patterns, rearfoot strike and forefoot strike, with three slope conditions: −6°, 0°, and +6°. Plantar pressure data were collected using a pressure measurement insole. At the heel region, peak pressure in rearfoot strike increased by approximately 32.1% during downhill running and decreased by approximately 24.3% during uphill running compared to level running. At the forefoot region, regardless of foot strike pattern, peak pressure decreased by approximately 6.8% during downhill running compared to level running, with no significant differences between uphill and level running. Finally, Legge et al., in their perspective article, addressed the lack of a clear definition and guidelines regarding the sport-specific movement competency required for safe and effective rowing, particularly in the context of performance enhancement. They argued that movement competency should be emphasized alongside the physiological and biomechanical attributes of rowing performance. Based on the literature, they proposed a definition and examples highlighting that rowers must coordinate different regions of the body through appropriate joint positioning and movement patterns to safely optimize force development capacity during the stroke cycle. Regarding the association between muscle asymmetry and musculoskeletal disorders, Mao et al. investigated improvements in sagittal and coronal plane posture in a female patient with adolescent idiopathic scoliosis (AIS) and associated pain symptoms following a multidisciplinary comprehensive therapeutic intervention. A full-spine x-ray examination revealed cervical kyphosis, forward head posture (FHP), uneven shoulder height (left higher than the right), and right-convex thoracolumbar scoliosis with a Cobb angle of 40°. After 50 sessions over 32 weeks, the patient's postural deformity showed marked improvement. FHP decreased significantly, thoracic positioning became more centered, shoulder leveling was restored, the right-convex thoracolumbar scoliosis Cobb angle decreased by 7° in the thoracic region and 17° in the lumbo-sacral region, spinal rotation angle improved, and low back pain was greatly alleviated. Additionally, Yang et al. investigated how abnormal curvature influences the performance of the upper trapezius (UT) and sternocleidomastoid (SCM) during movement and their correlations. Cervical lateral radiographs measured the C2-C7 Cobb angle, categorizing participants into an abnormal curvature group (25 chronic neck pain patients) and a normal curvature group (24 healthy individuals). Surface electromyography (sEMG) monitored the UT and SCM during six cervical movements at maximal voluntary contraction. Abnormal cervical lordosis significantly increased dynamic muscle fatigue, manifesting as deteriorated sEMG spectral characteristics in the UT and SCM. Furthermore, a complex relationship exists between the cervical Cobb angle and muscle fatigue, confirming the hypothesis that abnormal curvature induces compensatory muscle activation. Finally, this research topic also included articles from the field of sports diagnostics, such as the study by Toskić et al. In a sample of 158 individuals, they investigated the relationships between asymmetry indices derived from isokinetic dynamometry (ID) and tensiomyography (TMG). Pearson correlation analysis revealed no significant correlations between ID and TMG in functional asymmetry and only small, inconsistent correlations for lateral asymmetry. The findings indicate that ID and TMG measure different dimensions of muscle asymmetry and therefore should not be considered interchangeable. While TMG provides valuable information on peripheral contractile properties, it does not appear to replicate the joint-level asymmetry outcomes obtained with ID and thus may not serve as a direct substitute for isokinetic assessment when evaluating lower-limb muscle asymmetries.

Hopefully, the presented studies will contribute to a better understanding of the influence of muscle asymmetry on movement performance and sports-related injuries, and prove useful for future research in this field.

